# Eplerenone Attenuates Pulse Wave Reflection in Chronic Kidney Disease Stage 3–4 - A Randomized Controlled Study

**DOI:** 10.1371/journal.pone.0064549

**Published:** 2013-05-21

**Authors:** Lene Boesby, Thomas Elung-Jensen, Svend Strandgaard, Anne-Lise Kamper

**Affiliations:** 1 Department of Nephrology, Herlev Hospital, University of Copenhagen, Herlev, Denmark; 2 Department of Nephrology, Rigshospitalet, University of Copenhagen, Copenhagen, Denmark; Universidade de São Paulo, Brazil

## Abstract

**Background:**

Patients with chronic kidney disease (CKD) have high cardiovascular mortality and morbidity associated with increased arterial stiffness. Plasma aldosterone levels are increased in CKD, and aldosterone has been found to increase vascular inflammation and fibrosis. It was hypothesized that aldosterone receptor inhibition with eplerenone could reduce arterial stiffness in CKD stage 3–4.

**Study Design:**

The design was randomized, open, parallel group. Measurements of arterial stiffness markers were undertaken at weeks 1 and 24.

**Intervention:**

24 weeks of add-on treatment with 25–50 mg eplerenone or standard medication.

**Outcomes:**

Primary outcome parameter was carotid-femoral pulse wave velocity (cfPWV). Secondary outcomes were augmentation index (AIx), ambulatory arterial stiffness index (AASI) and urinary albumin excretion.

**Results:**

Fifty-four CKD patients (mean eGFR 36 mL/min/1.73 m^2^, SD 11) were randomized. Forty-six patients completed the trial. The mean difference in cfPWV changes between groups was 0.1 m/s (95%CI: −1.0, 1.3), P = 0.8. The mean difference in AIx changes between groups was 4.4% (0.1, 8.6), P = 0.04. AASI was unchanged in both groups. The ratio of change in urinary albumin excretion in the eplerenone group compared to the control was 0.61 (0.37, 1.01), P = 0.05. Four patients were withdrawn from the eplerenone group including three because of possible side effects; one was withdrawn from the control group. Mild hyperkalemia was seen on three occasions and was easily managed.

**Limitations:**

The full planned number of patients was not attained. The duration of the trial may have been too short to obtain full effect of eplerenone on the arteries.

**Conclusions:**

Add-on treatment with eplerenone in CKD stage 3–4 did not significantly reduce cfPWV. There may be beneficial vascular effects leading to attenuated pulse wave reflection. Treatment was well-tolerated.

**Trial Registration:**

ClinicalTrials.gov NCT01100203

## Introduction

Arterial stiffness is increased in patients with chronic kidney disease (CKD) compared to the background population [1]. This adds to the increased burden of cardiovascular morbidity and mortality in CKD. Aortic stiffness reversibility in response to blood pressure (BP) lowering has been shown to have a beneficial impact, which is independent of level of BP lowering on the survival of chronic haemodialysis patients and provides a potential treatment target [2]. Markers of arterial stiffness have been found to be independent predictors of all-cause and cardiovascular mortality in patients with CKD and hypertension as well as in the general population [3]–[10]. Attenuation of arterial stiffness has been achieved by treatment with blockers of the renin-angiotensin system (RAS) in patients with diabetes, hypertension and end stage renal disease (ESRD) [2], [11]–[14].

Aldosterone has pro-inflammatory and pro-fibrotic effects in extra-renal tissues including blood vessels and increased plasma levels of aldosterone are found in patients with CKD [15]–[17]. In rats supplied with aldosterone and salt, attenuation of aortic stiffness was reported after treatment with the selective aldosterone receptor blocker eplerenone [18]. Studies in human hypertension on resistance vessels and carotid-femoral aortic pulse wave velocity (cfPWV) have reported decreased vessel stiffness following treatment with eplerenone [19], [20]. A recent study in patients with CKD stage 2–3 reported a decrease of arterial stiffness after treatment with spironolactone, a non-selective aldosterone inhibitor added on to RAS-blockade and compared to placebo [21].

The primary aim of the present study was to test the effect of 24 week add-on eplerenone in CKD stage 3–4 on arterial stiffness parameters.

### Ethics Statement

The study was approved by the Danish Data Protection Agency, the Ethical Committee of the Capital Region of Denmark and the Danish Medicines Agency. The study was registered in the www.clinicaltrials.gov database, registration number NCT01100203. The study was carried out according to the Helsinki Declaration and written informed consent was obtained from all patients prior to inclusion.

## Subjects and Methods

### Design

The study was carried out in a randomized, open-label, parallel group design comparing an intervention group receiving eplerenone with a control group over a 24 week period. Randomization was done by the GCP-unit, University of Copenhagen.

### Study Participants

Before inclusion, patients were screened by blood samples and pulse wave measurements. Kidney function (eGFR) was evaluated by the CKD-EPI formula [22]. Inclusion criteria were age 18 to 80 years; eGFR 15–59 mL/min/1.73 m^2^; untreated BP>130/80 mmHg or use of anti-hypertensive drugs. Exclusion criteria were: plasma (p−) potassium >5.0 mEq/L; allergy to aldosterone antagonists; chronic liver insufficiency; ongoing treatment with CYP3A4-inhibitors, lithium or immunosuppressive agents including steroids; invalidating psychiatric disorders; other severe non-renal disease; implantations of vascular stents in the aorta, brachial or radial arteries; non-sinus rhythm; immeasurable pulse amplitude; limb amputations; woman of childbearing potential not using approved contraception; pregnancy or breast-feeding. Withdrawal criteria were pregnancy despite contraception during the study period; serious non-renal disease, and p–potassium >5.5 mEq/L persisting at extra control visits within one week, despite dietary instructions or treatment with furosemide; increase of p-creatinine above 30% from baseline value at two consecutive visits; unacceptable side effects; and wish for withdrawal from the participant or non-compliance. Patients were recruited from two outpatient clinics and all patients were seen by the principal investigator.

### Study protocol

Data collection at baseline included history of cardiovascular disease (CVD), previous and current smoking habits, medication and demographics. Patients were seen at week 1, 2, 4, 8, 12, 16, 20 and 24. In the eplerenone group, treatment was administered as add-on to ongoing therapy. The dose was 25 mg once daily for the first week and raised to 50 mg once daily for the remaining 23 weeks. The BP goal was <130/80 mmHg. In case of symptomatic hypotension, reductions in antihypertensive therapy were primarily made in non-RAS-blocking agents and in case of BP above target non-RAS-blocking agents were added. In case of hyperkalaemia (p-potassium >5.5 mEq/L), patients were given dietary instructions and increased doses of furosemide and were subsequently seen at extra control visits.

The protocol for this trial and supporting CONSORT checklist are available as supporting information; see Checklist S1 and Protocol S1.

### Outcome parameters

The primary endpoint was change in cfPWV after 24 weeks. Secondary endpoints derived from pulse wave analysis (PWA) were changes in augmentation indices: AIx and AIx@HR75 (see methods for description), and ambulatory arterial stiffness index (AASI) derived from 24 hour (24 h) ambulatory blood pressure measurements (ABPM). Other study parameters comprised albuminuria based on 24 h urine samples collected at week 1 and 24. Office BP, p-potassium, p-creatinine and eGFR were measured at every visit and applied as safety parameters.

### Methods

#### Pulse wave velocity and pulse wave analysis

The SphygmoCor® hardware and software (version 8.2, Atcor Medical, Sydney, Australia) and an applanation tonometer containing a high-fidelity micro-manometer (Millar SPT-301 tonometer, Houston, Texas, USA) were used for measurement of cfPWV and PWA as described elsewhere [23]. The recordings of the peripheral wave form were accepted if the variations in pulse height, diastole and pulse length were equal to or less than 7% as calculated by the software and the mean pulse height was above 80 mV. A quality index provided by the software was to be 80% or above. All recordings were made after 10 minutes of rest in a supine position, and were made in duplicate by the same trained observer on the right side of the patients.

The cfPWV was measured by recording the pulse wave at the site of the femoral artery followed by recording at the carotid artery along with simultaneous electrocardiograph recordings (ECG). The distance on the surface of the patient was measured from the suprasternal notch to the carotid and femoral arteries respectively and measure points were marked to ensure equal lengths of travel for the pulse wave at duplicate measurements made on the same day. The algorithm in the software subtracts the distances and PWVs are calculated with reference to the ECG. The SphygmoCor® software provides a validated generalized transfer function which performs all calculations of pulse wave measurements obtained through the applanation tonometer. The PWA included assessment of the augmentation index (AIx). The AIx is the difference between the second (P2) and the first (P1) systolic peaks expressed as a percentage of the pulse pressure (PP) and calculated as: ((P2-P1)/PP)*100. The AIx is dependent on heart rate (HR) and is therefore also reported as a value standardized to a HR of 75 beats per minute (AIx@HR75). HR at the pulse wave measurements as well as 24 h HR was recorded.

For the calibration of the pressure wave form in the general transfer function systolic and diastolic BP was measured manually as described below.

All measurements took place in the morning at 8 or 9 a.m. and patients were without intake of morning medication or food/drink.

#### Blood pressure measurements

Each recorded BP was based on three measurements after 10 minutes of rest. The first measurement was discarded and the average of the last two was used. Office BPs at the control visits were made with patients in the sitting position and at the pulse wave examination visits with patients in the supine position. Cuff width was selected according to arm circumference. All measurements were made by the principal investigator using sphygmomanometry.

24 h oscillometric ABPMs were obtained after measurement of pulse wave parameters. ABPM was made on the non-dominant arm with SpaceLabs 90217 equipment. Recorders were programmed to obtain measurements every 15 minutes during the daytime (8.00–23.00 hours) and every 30 minutes during the night (23.00 to 8.00 hours).

#### Ambulatory arterial stiffness index

For calculation of the AASI, the regression slope of diastolic on systolic BPs was computed for each individual 24 h BP recording and AASI was calculated as 1 minus the regression slope [4]. A minimum of ten recordings during daytime and five during night time were required.

#### Intervention

Dose titration of eplerenone from an initial 25 mg daily to 50 mg daily as maximal dose is recommended in the Summary of Product Characteristics (SPC). The dose was chosen after considerations on safety regarding potential risk of hyperkalaemia in CKD stage 3–4, as well as the intention of studying BP independent effects of eplerenone rather than reduction in arterial stiffness due to haemodynamic changes.

#### Blood and urine analyses

All biochemical analyses were carried out at the Department of Clinical Chemistry at Herlev Hospital.

#### Statistics

A sample size of 37 patients in each group was estimated according to the following criteria: a power set at 0.8, the level of significance at 0.05 and a minimal relevant difference in cfPWV between the groups of 1 m/s and a standard deviation (SD) of 1.5 m/s. Characteristics at baseline were compared by independent samples t-test for continuous variables and Fisher's exact test for dichotomous variables. Changes in outcome parameters were evaluated including only patients who completed the full 24 weeks by independent samples t-test.

Multiple linear regression analysis was applied to compare the changes in the two groups with adjustment for the baseline values of the particular variable (as recommended by Vickers [24]). Finally additional multiple regression analyses were carried out to investigate the influence of parameters known to influence arterial stiffness.

Laboratory data were pooled for control visits in order to investigate differences between groups during the stable treatment regime.

Data are presented as mean with standard deviations (SD) or with 95% confidence intervals (95%CI) for mean. Urinary albumin excretion was skewed and therefore log-transformed prior to analysis. Baseline data are presented as geometric means with 95%CI, and changes are presented as ratios with 95%CI. Data were analyzed using SPSS statistical software, version 20.

## Results

Patients were recruited consecutively from April 2010 through June 2011.

### Patient characteristics

**Figure 1:**
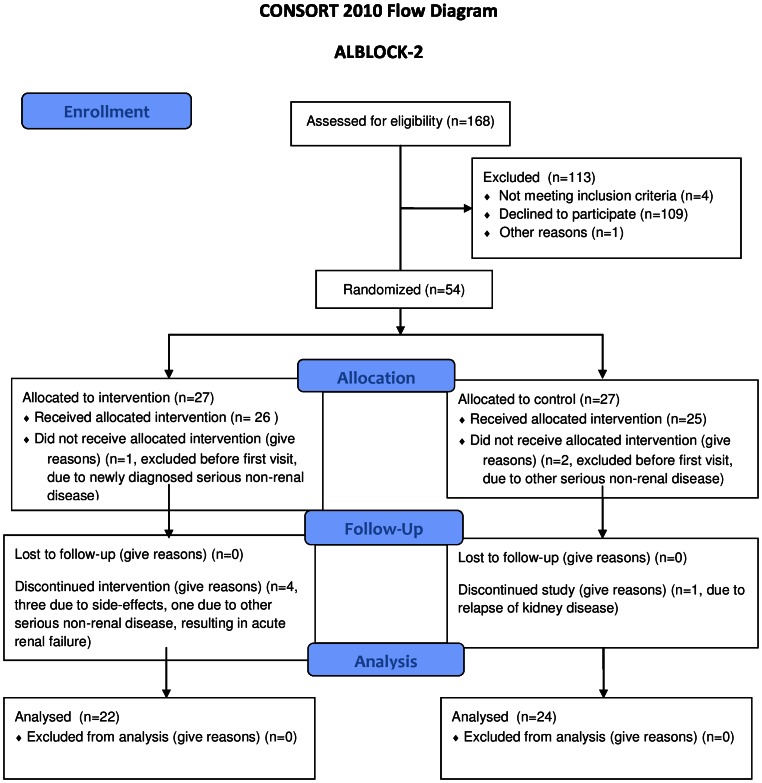
CONSORT FLOW DIAGRAM showing recruitment of patients and flow through study.

Fifty-four patients were included, and 46 completed the study. Three patients were withdrawn by the investigator prior to the first visit due to unexpected serious non-renal disease (one in the treatment group and two controls). Five patients did not complete the study, four in the eplerenone group and one in the control group. In the eplerenone group, three patients were withdrawn due to possible side effects: one due to recurrence of gout, one due to a feeling of swollenness of the tongue and one patient due to nausea, dizziness and general discomfort. The last withdrawal in the eplerenone group was caused by other serious non-renal disease resulting in acute renal failure. In the control group, one patient was withdrawn due to a relapse of glomerulonephritis. This left 22 patients in the eplerenone group and 24 patients in the control group (Figure 1). Unfortunately, it proved not possible to recruit the full planned number of patients within the time frame and resources allotted to the study. There were no differences in demographic data, and baseline values of vascular and laboratory data were comparable between the groups (Table 1 and Table 2).

**Table 1:** Demographics – baseline characteristics.

	Eplerenone group N = 26	Control group N = 25
**Males (%)**	19 (73)	19 (76)
**Age (years), mean±SD**	58.3±13.4	58.5±12.8
**Smoking current (%)**	2 (8)	4 (16)
**Diabetes (%)**	7 (27)	6 (24)
**Previous CVE** ∧ ** (%)**	6 (23)	4 (16)
**Weight (kg), mean±SD**	89.1±19.9	90.5±15.0
**Anti-hypertensive medication (%)**	26 (100)	25 (100)
Number of anti-hypertensive drugs, median (IQR)	2 (2–3)	3 (2–4)
ACE-inhibitor	13 (50)	14 (56)
Angiotensin Receptor Blocker	10 (38)	13 (52)
Combined ACE-inhibitor and Angiotensin Receptor Blocker	0 (0)	4 (16)
RAS-blockade – total	23 (88)	23 (92)
Calcium Channel Blocker	11 (42)	10 (40)
Beta Blocker	8 (31)	12 (48)
Furosemide	18 (69)	13(76)
Diuretic – other	4 (15)	5 (20)
**CKD stage 3/4**	18/8	15/10
**Renal diagnosis**		
Chronic glomerulonephritis	5	5
Vascular disease	3	1
ADPKD	6	4
Diabetic nephropathy	0	2
Other	1	5
Unknown	11	8

ACE, angiotensin converting enzyme; ADPKD, autosomal dominant polycystic kidney disease; CKD, chronic kidney disease; CVE, cardiovascular event, i.e. acute myocardial infarction (MI) or stroke (ischaemic or haemorraghic); IQR, inter quartile range; RAS, renin-angiotensin-system.

∧ No patients had had both stroke and MI.

All P-values >0.05.

**Table 2:** Baseline vascular and laboratory values.

	Eplerenone group N = 26	Control group N = 25
	Mean (SD)	Mean (SD)
**Indices of arterial stiffness**		
cfPWV (m/s)	10.1 (4.0)	9.8 (3.3)
AIx (%)	22.3 (11.1)	24.6 (9.4)
AIx@HR75 (%)	16.7 (10.3)	19.2 (7.9)
AASI (units)	0.47 (0.12)	0.46 (0.14)
**Blood pressure data**		
systolic office BP (mmHg)	126 (15)	129 (15)
diastolic office BP (mmHg)	77 (8)	79 (11)
central systolic BP (mmHg)	116 (14)	119 (15)
central diastolic BP (mmHg)	78 (8)	80 (11)
24 h systolic BP (mmHg)	126 (10)	127 (17)
24 h diastolic BP (mmHg)	73 (8)	75 (10)
**Renal data**		
P-creatinine (mg/dL)	1.97 (0.53)	2.09 (0.63)
P-urea (mg/dL)	37.8 (14.6)	39.2 (15.1)
P-potassium (mEq/L)	4.3 (0.4)	4.3 (0.5)
P-bicarbonate (mEq/L)	24 (3)	25 (3)
eGFR (mL/min/1.73 m^2^)	36 (10)	35 (13)
Creatinine clearance (mL/min)	54 (23)	55 (26)
Urinary albumin (mg/24 h)*	241 (121, 479)	208 (89, 486)

*Note:* Conversion factors for units: p-creatinine in mg/dL to µmol/L, ×88.4; p-urea in mg/dL to mmol/L, ×0.357. No conversion necessary for p-potassium or p-bicarbonate in mEq/L and mmol/L.

*AASI, Ambulatory Arterial Stiffness Index; AIx, Augmentation Index; AIx@HR75, Augmentation Index adjusted for heart rate 75 beats/minute; BP, blood pressure; cfPWV, carotid-femoral pulse wave velocity; eGFR, estimated glomerular filtration rate;*

Indices of arterial stiffness are a mean of two measurements per patient, except AASI.

* 24 h urinary albumin excretion is presented as geometric means with 95% CI.

All P-values>0.05.

### Quality of measurements

All pulse wave measurements and ABPMs fulfilled the quality requirements.

Table 3 shows mean values with SDs after 24 weeks of the various outcome parameters in the eplerenone and control groups. Table 4 shows the mean differences in changes between groups with 95% CI and P-values based on the multiple regression model where the baseline values and adherence to eplerenone or control group were entered as co-variates. Multiple regressions with additional co-variates are shown in Table S1.

**Table 3:** Vascular and laboratory values after 24 weeks.

	Eplerenone group N = 22	Control group N = 24
	Mean (SD)	Mean (SD)
**Indices of arterial stiffness**		
cfPWV (m/s)	9.5 (2.9)	9.4 (3.2)
AIx (%)	20.4 (11.4)	27.3 (8.7)
AIx@HR75 (%)	14.6 (9.6)	21.3 (7.6)
AASI (units)	0.45 (0.16)	0.45 (0.15)
**Blood pressure data**		
systolic office BP (mmHg)	125 (14)	131 (16)
diastolic office BP (mmHg)	77 (9)	81 (12)
central systolic BP (mmHg)	115 (15)	122 (16)
central diastolic BP (mmHg)	78 (9)	82 (13)
24 h systolic BP (mmHg)	122 (10)	125 (15)
24 h diastolic BP (mmHg)	72 (9)	74 (11)
**Renal data**		
P-creatinine (mg/dL)	2.21 (0.67)	2.13 (0.60)
P-urea (mg/dL)	45.4 (20.7)	40.9 (21.3)
P-potassium (mEq/L)	4.6 (0.6)	4.4 (0.6)
P-bicarbonate (mEq/L)	24 (4)	24 (3)
eGFR (mL/min/1.73 m^2^)	33 (10)	34 (13)
Creatinine clearance (mL/min)	50 (19)	54 (28)
Urinary albumin (mg/24 h)	137 (67, 280)	178 (76, 417)
(%)*	−40 (−50, −27)	0 (−38, 58)

*Note:* Conversion factors for units: p-creatinine in mg/dL to µmol/L, ×88.4; p-urea in mg/dL to mmol/L, ×0.357. No conversion necessary for p-potassium or p-bicarbonate in mEq/L and mmol/L.

*AASI, Ambulatory Arterial Stiffness Index; AIx, Augmentation Index; AIx@HR75, Augmentation Index adjusted for heart rate 75 beats/minute; BP, blood pressure; cfPWV, carotid-femoral pulse wave velocity; CI, confidence interval; eGFR, estimated glomerular filtration rate;*

Indices of arterial stiffness are a mean of two measurements per patient, except AASI.

* 24 h urinary albumin excretion is presented as geometric means with 95% CI and ratio in percent.

Effect size, Δ Eplerenone – Δ Control, is given as ratio between eplerenone group and control group.

**Table 4:** Mean differences between eplerenone and control group after 24 weeks adjusted for baseline values.

	Δ Eplerenone – Δ Control, (adjusted)	P-values
	Mean (95% CI)	
**Indices of arterial stiffness**		
cfPWV (m/s)	0.1 (−1.0,1.3)	0.8
AIx (%)	4.4 (0.1, 8.6)	0.04
AIx@HR75 (%)	3.8 (0.3, 7.4)	0.04
AASI (units)	0.01 (−0.08, 0.09)	0.9
**Blood pressure data**		
systolic office BP (mmHg)	4 (−3, 11)	0.2
diastolic office BP (mmHg)	3 (−1, 7)	0.1
central systolic BP (mmHg)	5 (−2, 13)	0.2
central diastolic BP (mmHg)	3 (−1, 7)	0.1
24 h systolic BP (mmHg)	3 (−2, 8)	0.2
24 h diastolic BP (mmHg)	2 (−2, 5)	0.4
**Renal data**		
P-creatinine (mg/dL)	−0.19 (0.38, 0.00)	0.05
P-urea (mg/dL)	−8.4 (−16.8, −2.8)	0.1
P-potassium (mEq/L)	−0.2 (−0.5, 0.0)	0.1
P-bicarbonate (mEq/L)	0 (−2, 2)	0.9
eGFR (mL/min/1.73 m^2^)	3 (−1, 6)	0.2
Creatinine clearance (mL/min)	4 (−3, 11)	0.3
Urinary albumin (%)*	0.61 (0.37, 1.01)**	0.05

*Note:* Conversion factors for units: p-creatinine in mg/dL to µmol/L, ×88.4; p-urea in mg/dL to mmol/L, ×0.357. No conversion necessary for p-potassium or p-bicarbonate in mEq/L and mmol/L.

*AASI, Ambulatory Arterial Stiffness Index; AIx, Augmentation Index; AIx@HR75, Augmentation Index adjusted for heart rate 75 beats/minute; BP, blood pressure; cfPWV, carotid-femoral pulse wave velocity; CI, confidence interval; eGFR, estimated glomerular filtration rate;*

Indices of arterial stiffness are a mean of two measurements per patient, except AASI.

* 24 h urinary albumin excretion is presented as geometric means with 95% CI and ratio in percent.

** Effect size, Δ Eplerenone – Δ Control, is given as ratio between eplerenone group and control group.

Multiple linear regression analysis was applied to compare the changes in the two groups with adjustment for the baseline values of the particular variable.

### Pulse wave velocity and pulse wave analysis

The mean change in cfPWV during the study was −0.9 m/s (−1.9 to 0.1), N = 22, in the eplerenone group and −0.6 m/s (−1.5 to 0.3), N = 24, in the control group. The mean difference between changes in the groups was 0.1 m/s (−1.0, 1.3), P = 0.8 with adjustment for baseline values. Adjustment for values of systolic BP, HR and diabetic status did not alter this finding. Mean AIx for patients in the two groups together was 22% (19, 26) for men and 27% (21, 32) for women. The mean change in AIx during the study was −0.3% (−3.7, 3.2) in the intervention group and in the control group it was 3.2% (0.5, 5.8). The difference between changes in the groups was 4.4% (0.1, 8.6), P = 0.04 in favour of eplerenone.

### Ambulatory arterial stiffness index

There was no significant difference in changes of AASI between the groups.

### Blood Pressure and heart rate

The 24 h systolic BP fell in the eplerenone group by 4.7 mmHg (−8.6, −0.8), and in the control group by 1.3 mmHg (−5.5, 3.0). The difference between changes in the groups was 3 mmHg (−2, 8), P = 0.2. The 24 h diastolic BP, office systolic and diastolic BPs, central BPs, as well as office BPs at control visits did not differ significantly between the eplerenone and control group (Table 3). Mean HR at baseline was 63 beats/min (59, 67), in the eplerenone group and 64 (58, 69) in the control group, P = 0.8. There were no significant changes between groups, P = 0.4. Mean 24 h HR at baseline was 71 beats/min (66, 75) in the eplerenone group and 69 (65, 72), in the control group, P = 0.4. There were no significant changes between groups, P = 0.07.

### Safety parameters

The mean values of p-potassium, p-creatinine and eGFR for the control visits did not differ significantly between the groups. Increases were seen during eplerenone treatment in p-potassium and p-creatinine, but changes were not significant (Table 4). The change in creatinine clearance was not different between the two groups, P = 0.3. In total three measurements of p-potassium were above 5.5 mEq/L in the eplerenone group, maximum value 5.7 mEq/L, while in the control group two measurements of p-potassium were above 5.5 mEq/L, maximum value 5.6 mEq/L. During treatment mean p-potassium was 4.6 mEq/L (4.4, 4.7) in the eplerenone group and 4.4 mEq/L (4.3, 4.6) in the control group. The difference between changes in the groups was −0.2 mM (−0.5, 0.0), P = 0.2.

### 24 h Urine collections

The mean change in urinary albumin excretion in the intervention group was −40% (−50, −27), while in the control group it was unchanged, 0% (−38%, 58%). The ratio of change in urinary albumin excretion in the eplerenone group compared to the control group was 0.61 (0.37, 1.01), P = 0.05 indicating a relative decrease in 24 h urinary albumin excretion of 39% (63%, −1%) in the eplerenone group compared to the control group. Adjustments for differences in BP and renal function (eGFR or creatinine clearance), did not alter this.

## Discussion

The present study shows that 24 weeks add-on of the selective aldosterone receptor inhibitor, eplerenone, in CKD stage 3–4 had no effect on cfPWV, but caused a significant effect on the pulse wave reflection judged by AIx and AIx@HR75, which tended to decrease in the eplerenone group and increased in the control group.

The importance of changes in AIx in patients with ESRD has previously been demonstrated. It was reported, that for every 10% absolute increase in AIx the risk of all-cause mortality increased by 1.51 and CV mortality by 1.48 [10]. A similar result was found in pre-dialysis patients [25]. Thus prevention of increase in AIx, as shown in the present study, may have important prognostic implications.

cfPWV was the same order of magnitude as in a previous study by our group in CKD patients of similar age [26], and in both studies cfPWV was higher than in healthy individuals of the same age (10 m/s vs. 8 m/s) [27]. By contrast the baseline AIx levels in the present study are of the same order of magnitude as found in healthy individuals of comparable age [27], and is somewhat lower than in our earlier study [26], possibly because of better BP control and treatment with RAS-blockers in almost all patients. Still, the elevated cfPWV demonstrates conduit artery remodelling with room for improvement by therapeutic intervention. In clinical studies eplerenone has been found to reduce stiffness in resistance arteries and in the aorta in newly diagnosed hypertensive patients with normal kidney function and in elderly hypertensive patients [19], [20]. Thus, treatment for one year with 50 mg eplerenone daily reduced the in vitro stiffness in resistance vessels in gluteal biopsies when compared to treatment with the beta blocker atenolol [19]. In another study, the calcium channel blocker amlodipine and eplerenone both caused significant reduction of cfPWV over 24 weeks, but the patients had very high baseline BPs and cfPWVs. Data on pulse wave reflection and kidney function were not reported [20]. A study in young normotensive, drug-free patients with autosomal dominant polycystic kidney disease and normal kidney function showed an increased AIx, whereas cfPWV was the same as in a normal control group [27]. It was proposed, that AIx was higher than expected according to age and kidney function due to early-onset changes in small resistance vessels, preceding hypertensive and uremic remodelling in the larger conduit vessels. This is in line with our results and Savoia et al [19] where the lowering of AIx may be a result of eplerenone facilitating a reduction in peripheral resistance, which has seemingly not lost its adaptive abilities. A parallel group, placebo controlled study by Edwards et al in 112 patients with CKD stage 2–3 reported significant reductions in cfPWV (Δ0.8 m/s), and AIx (Δ 5.2%) versus ΔcfPWV 0.1 m/s and ΔAIx 1.4% in the control group after 40 weeks of treatment with spironolactone [21]. Speculations on reasons for our results differing from those of Edwards et al [21] could be differences in pharmacological actions between eplerenone and spironolactone as well as shorter intervention time and a greater disease burden in the present study.

In the present study there were no changes in AASI, peripheral (office and 24 h) or central BP. Within the eplerenone group both 24 h systolic and diastolic BPs were reduced, though the change was only found to be significant for systolic BP. This may explain why AASI was not changed, since the two parameters changed in the same direction, and AASI is calculated from the regression line of diastolic on systolic BP. It is of note, that the 24 h systolic BP is almost identical to the office BP. This confirms a previous finding by finding by our group [28], but is at variance with other studies, where 24 h systolic BPs are normally found to be lower than office BPs [28], [29]. An explanation for this could be the lack of night time dipping in patients with CKD [28], [30].

The increase in arterial stiffness is a result of changes in the extracellular matrix as well as the contractile elements in the vessel wall. These processes are accelerated in CKD and ESRD, and may in part be due to increased levels of aldosterone [31]. P-aldosterone levels are increased in CKD. This applies to those who are naïve to treatment with RAS-blockers as well as to those in such treatment [17], [32]. Animal studies have shown that aldosterone induces inflammation leading to vascular fibrosis and that blockade of mineralocorticoid receptors reduce this response [18].

The present study also investigated 24 h urinary albumin excretion, which was found in a previous study to be decreased during 8 weeks of treatment with eplerenone [33]. After 24 weeks the 24 h urinary albumin excretion was lowered by 40% in the eplerenone group, but unchanged in the control group. This difference did not reach statistical significance.

Safety evaluation showed increases in p-potassium and p-creatinine, as well as a decrease in eGFR, but there were no significant differences when compared with control patients and generally, the treatment with eplerenone 50 mg once daily was well tolerated. Despite this, it is important that patients with reduced renal function have potassium supplements withdrawn and p-potassium monitored frequently when treatment with eplerenone is initiated.

### Limitations

The main limitation is that the number of patients needed according to power calculations was not obtained. The closure of the study was in no manner related to results or interim analysis. The study was planned within a fixed time frame which it was not possible to prolong. Power calculations were based on expected difference in cfPWV. Therefore there may be risk of a type 2 error concerning the lack of effect on that parameter. The difference in AIx between the eplerenone and control groups was significant, making sample size less of an issue. The duration of treatment time may have been inappropriate for remodelling of the conduit arteries, or the arterial stiffness may have been too advanced to respond fully to eplerenone.

## Conclusions

Add-on treatment with eplerenone in CKD stage 3–4 may have beneficial vascular effects leading to attenuated pulse wave reflection. The treatment was well-tolerated.

## Supporting Information

Table S1 **Regression analysis – supplementary material.** *AIx, augmentation index; AIx@HR75, augmentation index adjusted for heart rate 75 beats/min; B, estimate; CI, confidence interval; R^2^ , coefficient of determination.* **Model 1** is value adjusted for baseline of the appropriate parameter. **Model 2** further adjusted for baseline cfPWV.
(DOC)

Checklist S1 **CONSORT Checklist.**
(DOC)

Protocol S1 **Trial Protocol.**
(DOCM)
