# Supplementary material for: Eplerenone Attenuates Pulse Wave Reflection in Chronic Kidney Disease Stage 3–4 - A Randomized Controlled Study
Source: PLoS One. 2013 May 21;8(5):e64549. doi: 10.1371/journal.pone.0064549 (PMC3660355; doi:10.1371/journal.pone.0064549)
Supplement: Table S1 — Regression analysis – supplementary material. AIx, augmentation index; AIx@HR75, augmentation index adjusted for heart rate 75 beats/min; B, estimate; CI, confidence interval; R2 , coefficient of determination. Model 1 is value adjusted for baseline of the appropriate parameter. Model 2 further adjusted for baseline cfPWV. (DOC) [file pone.0064549.s001.doc]

***Table S1*** Regression analysis – supplementary material

| **Parameter** | **Univariate** | | | **Model 1** | | | **Model 2** | | |
| --- | --- | --- | --- | --- | --- | --- | --- | --- | --- |
|  | ***R^2^*** | ***B (95%CI)*** | ***P*** | ***R^2^*** | ***B (95%CI)*** | ***P*** | ***R^2^*** | ***B (95%CI)*** | ***P*** |
| ΔAIx | 0.06 | 3.4 (-0.9, 7.8) | 0.12 | 0.16 | 4.4 (0.1, 8.6) | 0.04 | 0.16 | 4.3 (-0.1, 8.6) | 0.054 |
| ΔAIx@HR75 | 0.46 | 2.7 (-1.1, 6.4) | 0.16 | 0.20 | 3.81 6(0.3, 7.4) | 0.04 | 0.21 | 3.7 (0.0, 7.3) | 0.048 |

*AIx, augmentation index; AIx@HR75, augmentation index adjusted for heart rate 75 beats/min; B, estimate; CI, confidence interval; R^2^ , coefficient of determination*

**Model 1** is value adjusted for baseline of the appropriate parameter.

**Model 2** further adjusted for baseline cfPWV
