# Supplementary material for: Eplerenone Attenuates Pulse Wave Reflection in Chronic Kidney Disease Stage 3–4 - A Randomized Controlled Study
Source: PLoS One. 2013 May 21;8(5):e64549. doi: 10.1371/journal.pone.0064549 (PMC3660355; doi:10.1371/journal.pone.0064549)
Supplement: Protocol S1 — Trial Protocol. (DOCM) [file pone.0064549.s003.doc]

**Aldosterone receptor blockade in chronic kidney disease.**

**Influence on arterial stiffness and kidney function.**

**(ALBLOCK-2)**

**A translated copy of the approved protocol in Danish:**

ALBLOCK-2, version 11, 21 04 2010

**Sponsor/investigator Lene Boesby, MD**

**Investigator Thomas Elung-Jensen, MD Ph D**

**Investigator Svend Strandgaard, MD DMSc**

**Investigator Anne-Lise Kamper, MD DMSc**

**Table of contents**

[1. General information 3](#_Toc332279999)

[1.1 Sponsor-investigator is 3](#_Toc332280000)

[1.2 Locations and time frame 3](#_Toc332280001)

[1.3 Collaborators 3](#_Toc332280002)

[2. Background 3](#_Toc332280003)

[3. Study description 5](#_Toc332280004)

[3.1 Aim and hypothesis 5](#_Toc332280005)

[3.2 Design 5](#_Toc332280006)

[3.3 Endpoints 6](#_Toc332280007)

[3.4 Methods 6](#_Toc332280008)

[3.5 Randomization 8](#_Toc332280009)

[3.6 Study Population 8](#_Toc332280010)

[3.7 Inclusion criteria 9](#_Toc332280011)

[3.8 Exclusion criteria 9](#_Toc332280012)

[3.9 Withdrawal criteria 9](#_Toc332280013)

[3.10 Demographics 10](#_Toc332280014)

[3.11 Laboratory data 10](#_Toc332280015)

[3.12 Study equipment 12](#_Toc332280016)

[3.13 Practical performance of study 12](#_Toc332280017)

[4. Procedure for termination of the study 13](#_Toc332280018)

[5. Statistics 13](#_Toc332280019)

[7. Ethical considerations 14](#_Toc332280020)

[8. Adverse events and side effects 15](#_Toc332280021)

[9. Resume 15](#_Toc332280022)

[10. Information of the patient and informed consent 16](#_Toc332280023)

[11. Publication and reporting to authorities 17](#_Toc332280024)

[12. Insurance 17](#_Toc332280025)

[13. Economy 17](#_Toc332280026)

## General information

### 1.1 Sponsor-investigator is

Lene Boesby, MD, PhDstudent

Department of Nephrology

Herlev Hospital

Herlev Ringvej 75

DK-2730 Herlev

Denmark

Tel: +45 38 68 48 78

Fax: +45 38 68 37 09

E-mail: [lene@boesby.dk](mailto:lenboe03@heh.regionh.dk)

### 1.2 Locations and time frame

Department of Nephrology

Herlev Hospital

Herlev Ringvej 75

DK-2730 Herlev

Denmark

Study initiation is expected in Spring 2010 and completion by 1.3.2012. The study is complete when the last patient has completed the last visit. Recruitment rate is expected to be 1/week.

### 1.3 Collaborators

The GCP unit Copenhagen University

Bispebjerg Hospital, Building 51, 3rd floor

Bispebjerg Bakke 23

DK-2400 København NV

The study protocol and information material will be submitted for approval by the Danish Medicines Agency and the regional ethics committee for Region Hovedstaden. The study will be submitted to the [www.clinicaltrials.gov](http://www.clinicaltrials.gov) database before inclusion of the first participant.

## Background

The number of patients on chronic dialysis is increasing in Denmark and the rest of the Western part of the world (1;2). This is partly due to the fact that dialysis treatment is now also offered to patients with concomitant serious diseases. The prevalence as well as the incidence of end stage renal disease (ESRD) has been increasing in the past many years even if it seems that the incidence has been stabilised in the past 3 years in Denmark (2). It is only a limited part of patients with chronic kidney failure who are offered renal replacement therapy in the form of kidney transplants.

Patients with chronic kidney disease (CKD) have increased cardiovascular morbidity and mortality mainly due to arteriosclerosis even at young age. Therefore survival amongst patients on chronic dialysis is lower than for the normal population. It is essential to improve treatment for these patients in attempts to hinder or slow down progression of CKD to ESRD.

Almost all patients with CKD also suffer from moderate to severe hypertension. Treatment of hypertension is one of the cornerstones in treatment of CKD and hindrance of disease progression as well as reducing cardiovascular (CV) disease risk. It has been shown that the renin-angiotensin-aldosterone system (RAAS) plays a central role in progression of uraemia. Antihypertensive treatment that blocks the RAAS has specific renoprotective properties, independent of blood pressure (BP) reduction (3;4). The use of Angiotensin Converting Enzyme inhibitors (ACEi) and Angiotensin-2-receptorblockers (ARB) is nowadays standard in the treatment of CKD. Several studies have shown beneficial effect on CV morbidity and mortality in groups of patients with heart failure (5), hypertension (6), diabetic nephropathy (7) and non-diabetic nephropathy with proteinuria (8).

Furthermore there are studies who investigate an even more intense blockade of the RAAS, namely including an aldosterone inhibitor. There are studies showing a beneficial effect on survival of patients with heart failure (9;10). Furthermore triple blockade (ACEi, ARB and aldosterone inhibitor (spironolactone)) has an additive effect in reducing proteinuria, a surrogate marker of kidney function and disease progression, in patients with non-diabetic kidney disease (11;12).

It is proposed, that progression of renal insufficiency is multifactorial. Amongst these factors are vasoactive hormones, growth factors and cytokines (13). Besides the so-called classic (genomic) effects of aldosterone on fluid and potassium homeostasis, aldosterone also has non-genomic effects. Animal studies have suggested aldosterone to play a role in the development in hypertensive and arteriosclerotic disease in the heart and blood vessels (13;14).

Discussions whether it is the classical or non-classical effects of aldosterone that are responsible for these deleterious effects are still ongoing (15).

In patients with CKD another vessel disease is also present – the uraemic intima media stiffness. It differs in appearance from atherosclerosis in its universal form in both arteries and arterioles (16).

The vessels gradually change from elastic to very rigid formations increasing the overall load on the vessels. Arterial stiffness is an independent predictor for CV morbidity and mortality in the background population as well as in patients with CKD (17;18) and a reduction in the pulse wave velocity (PWV) can reduce morbidity and mortality in this group of patients (18).

In hypertensive patients with a serum creatinine < 300 µmol/L a positive and independent correlation between s-creatinine and aortic PWV has been found (19). Age, blood pressure and diabetes mellitus (DM) are other factors that independently influence PWV. A population study showed that PWV as well as the Augmentation Index (AIx) change with age. PWV is relatively constant up till the 8^th^ decade after which it tends to increase, whereas AIx rises through the first five decades after which it is relatively constant (20). Changes in the central and peripheral pulse wave parameters reflect changes in the vascular structure and function, which provide good evidence of changes in arterial stiffness. Several studies have been performed using the applanation tonometry method, where ACEi as well as ARBs have been found to reduce arterial stiffness (21;22). Treatment with ACEis and/or ARBs in CKD has been shown to be beneficial (23;24). When pulse wave measurements have been performed, AIx and other relevant values are derived, as well as the PWV.

Arterial stiffness is also depicted in the well known 24 hour ambulatory blood pressure measurement (AMBP). A quantitative measure for the stiffness is the Ambulatory Arterial Stiffness Index (AASI), derived from these measurements (25-30). AASI has been shown to be an independent predictor for stroke in the background population (27-29;31) and may be useful as a surrogate parameter for assessing the risk of CV disease (31). This has not been investigated for CKD.

The investigators of this study are currently examining the relationship between arterial stiffness as measured by applanation tonometry and the correlation to AASI in CKD stages 2-5 (ALBLOCK-1).

Results of combination therapy, in the form of dual blockade of the RAAS by ACEi and ARB, points towards a beneficial, additive effect on arterial stiffness in different patient populations. Studies to explore the effect of aldosterone inhibition on arterial stiffness assessed by applanation tonometry in patients with CKD have previously not been performed. A possible positive effect could bring this treatment modality into the awareness of doctors treating patients with CKD in an attempt to attenuate their overt risk of CV morbidity and mortality.

Arterial stiffness has been shown to be a modifiable factor during different treatment regimens in patients with essential hypertension(32-38). It has not been studied in CKD.

In this protocol – ALBLOCK-2 - intervention is the relatively new aldosterone receptor antagonist eplerenone. Eplerenone has fewer side effects than spironolactone, the previously single aldosterone blocker on the market. Prescription indications are at present heart failure after acute myocardial infarction or potassium sparing diuretic. It is known that aldosterone inhibition using spironolactone reduces albumin excretion in diabetic and in non-diabetic CKD(39). Besides this expected effect which has been studied in a very recently completed study by our group (data not published at present) eplerenone reduces stiffness in the smaller resistance vessels when evaluated by electro myograph in these vessels from patients with hypertension(40).

This effect has not been studied in CKD.

## Study description

### 3.1 Aim and hypothesis

The hypothesis of the ALBLOCK-2 study is that inhibition of aldosterone receptors using eplerenone in standard dosing will lead to a reduction in arterial stiffness in patients with different stages of CKD.

The aim is to reduce PWV by treating patients with eplerenone.

Secondarily it is studied whether AASI changes.

### 3.2 Design

The study is a prospective, randomized, controlled, parallel group, phase IV study.

| Week number | -4 | 1 | 2 | 4 | 8 | 12 | 16 | 20 | 24 |
| --- | --- | --- | --- | --- | --- | --- | --- | --- | --- |
| Visit number |  | 1a/1b | 2 | 3 | 4 | 5a/5b | 6 | 7 | 8a/8b |

A BP target of 120-130/80-90 is set and investigators will strive to keep this constant in patients throughout their enrolled period. Therefore a run-in period of 4 weeks has been planned. All patients are to continue on their normal medication including blockers of RAS, which most of these patients receive before enrolment. If anti-hypertensive treatment needs to be intensified, this is done by adding beta blocker, calcium antagonist or methyldopa. If BP is lower than target pressure medication will be withdrawn, primarily via reduction in calcium antagonism or beta blockade, while RAS-blockade in the initial form is aimed to be kept as it was.

If study participants fail to tolerate the full dose of eplerenone 50 mg once daily, dose will be reduced to 25 mg once daily. In case of the patients receiving potassium supplements at time of enrolment these will be withdrawn before start of intervention.

Participants are randomized after the run-in period and are given consecutive numbers of identification.

The study plan is identical for the two groups.

### 3.3 Endpoints

Primary endpoint is the difference in PWV measured by the tonometer (Millar, Houston, Texas, USA) and using the SphygmoCor® hard- and software.

Secondary endpoints are pulse wave morphology, evaluated by the AIx, BP, subendocardial viability ration (SEVR), heart rate, time to reflection (TR), ejection duration (ED), pulse pressure (PP), kidney function (estimated glomerular filtration rate (eGFR)), p-creatinine, AASI and albuminuria.

P-potassium and p-creatinine are used for safety monitoring.

### 3.4 Methods

*Pulse wave measurements*

Measurement of pulse wave is done by using applanation tonometry, which is non-invasive. Applanation tonometry provides information on the peripheral pulse wave morphology. The shape of the pulse wave is the sum of the forward going wave and the reflected wave. The reflected wave arises as a reflection of the incident wave form the periphery. In healthy individuals without atherosclerotic plaques or arteriosclerotic vessels the reflected wave returns in late systole or early diastole of the cardiac cycle. In people with arteriosclerosis the reflected wave returns earlier in systole, thereby compromising the filling of the coronary arteries and increasing the load on the ventricles leading to increased central blood pressure, left ventricular hypertrophy and increased risk of stroke and myocardial infarction. By using mathematical calculations in the generalized transfer function, built in in the SphygmoCor® device, information can be gained on the central blood pressure (aorta ascendens) from measurements performed on peripheral arteries (a. radialis). Validation of the pulse wave transfer function has been done invasively and it is now generally accepted as the gold standard for assessing arterial stiffness (41). Several studies have shown good reproducibility with acceptable inter- and intraobserver variation (42-45).

Pulse wave measurements are performed by the same, trained observer in the morning under standardized conditions. Before performing the actual measurements, marks are made on the skin of the patient over the part of the a. radialis, a. carotis, and a. femoralis where the pulse is most prominent. The suprasternal notch (SN) is used as reference point and is also marked. A tape measure is used to measure the distances between the SN and a. radialis, carotid artery and a. femoralis, respectively. Patients are then requested to rest in the supine position, in a quiet room of constant temperature for 10 minutes. The measurements are all undertaken on the right side. Patients have to be in the fasting state and have to refrain from smoking 8 hours before examination. Diabetics are allowed a light meal and all participants are allowed a glass of water. All medications including study medication are to be taken after examination. Quality of the measurements is evaluated partly by a visually acceptable curve of the peripheral pulse wave and partly by a maximum difference between 2 successive measurements of ≤7% and an average height of the wave of > 80 mV(42). Pulse wave measurements are done in two sets. The first is the pulse wave (PW) and after this the PWV. The AIx is derived from the PW. It is calculated as the percentage of augmentation (AG) of the pulse pressure (PP). On the recordings of the peripheral waves the PP, the first peak of the pulse wave (P1) and the second peak (P2) are marked. The augmentation is the difference between P1 and P2. And the AIx is this difference taken out of the PP. These calculations are all performed automatically by the SphygmoCor software. An acceptable Quality Index (QI) is > 80%. QI is calculated by the software from the other stated quality indicators (pulse height, max. 7 % difference between two measurements).

*Ambulatory blood pressure measurements*

AMBPs are automatically measured blood pressures during ordinary daily activities for a time period of approximately 24 hours. AMBP has been found reproducible in population studies (25-30), hypertensive populations (46) and diabetics (47). The reproducibility of AASI has been proven less reliable in an essentially hypertensive population (48) . The brachial BP is measured in the office using the ABPM equipment on the non-dominant arm. After manual measurements the apparatus will automatically measure the BP every 15 minutes in daytime, which is defined from 7 am until 11 pm. Hereafter the measurements will be performed every 30 minutes from 11 pm till 7 am (49). All patients receive a diary in which they are to make notes of physical activity, meals and rest as well as bedtime and rising time in the morning. For calculation of AASI a minimum of 21 readings of BP is required. These must be spread out over the whole period with at least 7 successful readings during night time. AASI is calculated in the following manner: (1-(the slope of diastolic BP/slope of systolic BP)). It follows that the stiffer the arterial tree, the more the regression line will go towards 0 and AASI thus towards 1. Previous studies have provided references for AASI in the background population. AASI for normotensive individuals is 0.57. In younger individuals AASI is 0.53 increasing to 0.72 in the elderly (30).

*Intervention – study medication*

The study medication is eplerenone, a selective aldosterone receptor antagonist. It is an approved drug, which in this study will be used on a non-appraised indication. The Summaries of Product Characteristics (SPC) in Danish will be used as reference document for evaluation of possible side effects and will be accessible at the site of investigation when the study is initiated.

Initial recommended dose is 25 mg once daily. The dose will be increased to 50 mg eplerenone in once daily dosing after 1 week. In this study eplerenone will be added to the study participants’ usual medication. The manufacturing company Pfizer Aps has been informed of the study.

*Control*

The control group will continue their usual medical therapy.

All dispensed study medication is registered in the participant’s CRF. At all visits compliance is noted and counts of tablets are made at the end of the study period. The study medication is provided by the hospital pharmacy in usual packages, with note of batch number and expiry date. Participants will be carefully instructed in the importance of compliance regarding study medication and participation as a whole.

Labels for study medication will contain the following information (in Danish):

*For use in clinical trial ALBLOCK-2*

*Patient no: ____ Visit no:___ No. tablets:______*

*Dose: 1 tablet daily. Should be taken in whole.*

*Unused medicine and empty packages to be returned to Lene Boesby*

*Sponsor-investgator: MD Lene Boesby, Dept. Nephrology, Herlev Hospital 44 88 48 78*

*Storage: At max 25°C out of reach of children.*

*In case of emergency please contact Lene Boesby*

*EudraCTno: 2009-012445-35*

3.5 Randomization

Randomization is done by blocks that are of unknown size to the investigators and sponsor by drawing of sealed, opaque envelopes provided by the GCP-unit of Copenhagen University.

At the time of randomization patients are given consecutive numbers of participation. Randomization will take place after the informed consent forms have been signed and the run-in period has been completed. Numbers will be noted in the participants’ CRF and in the patients’ medical file.

### 3.6 Study Population

Potential study participants are patients with CKD stages 3-4 (50), who at the time of recruitment are attending the outpatient clinics at the Departments of Nephrology at either Herlev Hospital or Rigshospitalet. Sponsor-investigator will be recruiting at both sites and all participants will be seen only at Herlev Hospital during the study period. Contact with potential participants will be made during a normal visit to the clinic or during admission to the ward.

Patients who fulfil inclusion criteria will be offered participation in the study. There is no financial compensation made in case of participation. Study medication is given free of charge.

### 3.7 Inclusion criteria

Patients are eligible when the following requirements are met:

- 18 years ≤ age ≤ 80 years
- voluntarily signed informed consent
- 15 ml/min/1,73 m^2^ ≤ eGFR < 60 ml/min/1,73 m^2^
- BP ≥ 130/80 mmHg or undergoing anti-hypertensive treatment

### 3.8 Exclusion criteria

Patients are ineligible for participation in the study if one of the following conditions is present:

- p-potassium is > 5.0 mM
- allergy to contents
- treated with spironolactone
- treated with potent inhibitors of CYP3A4 (see SPC for details)
- treated with lithium, ciclosporin, tacrolimus, prednisolone, or other immunosuppressing drug
- inborn errors of metabolism (see SPC for details)
- pregnancy or lactation
- fertile woman, not using safe contraception devices
- dementia or other psychiatric disorder, making understanding of the study conditions impossible
- other severe, chronic illness besides CKD, including liver insufficiency, according to investigators’ judgement
- vascular surgery including stenting or graftimplantation on a. brachialis, aorta or the carotid arteries
- systolic BP > 200 mmHg
- immeasurable pulse amplitude

### 3.9 Withdrawal criteria

Withdrawal form the study can be done if one of the following events occur:

- pregnancy
- other serious illness
- cardiovascular event such as myocardial infarction or stroke
- untreatable severe hyperkalaemia
- persisting increase in p-creatinine above 30% of base line value at first control visit
- unacceptable side effects
- participant’s wish to end participation before time
- doubts about the degree of compliance

Decision about withdrawal can be made by investigators. Patients withdrawn are not replaced in the study. In case of withdrawal, the patient will resume regular controls in outpatient clinics.

### 3.10 Demographics

The following demographic parameters are registered for each patient: sex, age, weight, height, BP, heart rate, eGFR, renal diagnosis, other diagnoses, medication, actual and former tobacco and alcohol use, previous CV events. A case report form (CRF) will be filled out for each patient. Laboratory results will be on printed laboratory forms and will be entered into a database from these. Data will be entered continuously.

### 3.11 Laboratory data

| ***Visit number*** | ***Time with reference to randomization for the individual patient*** | ***Blood tests*** | ***Other*** |
| --- | --- | --- | --- |
|  | Screening | b-haemoglobin, b-leukocytes, b-thrombocytes, p-bilirubin, p-ALAT, p-alkaline phosphatase, hsC-reactive protein, p-creatinine, p-urea, p-sodium, p-potassium, p-bicarbonate, p-glucose, p-albumin, p-calcium-ion, p-phosphate, p-uric acid, eGFR, p-cholesterol, p-triglyceride, p-HDL, P-LDL, p-parathyroid hormone (PTH) and 1,25-dihydroxy-cholecalciferol (vitamin D3), 25-OH-Vitamin D, p-renin og p-aldosterone.  p-HCG in fertile women. | ECG |
| 1a | Week 1 | b-haemoglobin, b-leukocytes, b-thrombocytes, p-bilirubin, p-ALAT, p-alkaline phosphatase, hsC-reactive protein, p-creatinine, p-urea, p-sodium, p-potassium, p-bicarbonate, p-glucose, p-albumin, p-calcium-ion, p-phosphate, p-uric acid, eGFR, p-cholesterol, p-triglyceride, p-HDL, P-LDL, p-PTH. | 24 hour urine sampling |
| 1b | Week 1 | return of AMBP device |  |
| 2 | Week 2 | b-haemoglobin, b-leukocytes, b-thrombocytes, p-bilirubin, p-ALAT, p-alkaline phosphatase, hsC-reactive protein, p-creatinine, p-urea, p-sodium, p-potassium, p-bicarbonate, p-glucose, p-albumin, p-calcium-ion, p-phosphate, p-uric acid, eGFR. |  |
| 3 | Week 4 | b-haemoglobin, b-leukocytes, b-thrombocytes, p-bilirubin, p-ALAT, p-alkaline phosphatase, hsC-reactive protein, p-creatinine, p-urea, p-sodium, p-potassium, p-bicarbonate, p-glucose, p-albumin, p-calcium-ion, p-phosphate, p-uric acid, eGFR |  |
| 4 | Week 8 | b-haemoglobin, b-leukocytes, b-thrombocytes, p-bilirubin, p-ALAT, p-alkaline phosphatase, hsC-reactive protein, p-creatinine, p-urea, p-sodium, p-potassium, p-bicarbonate, p-glucose, p-albumin, p-calcium-ion, p-phosphate, p-uric acid, eGFR. |  |
| 5a | Week 12 | b-haemoglobin, b-leukocytes, b-thrombocytes, p-bilirubin, p-ALAT, p-alkaline phosphatase, hsC-reactive protein, p-creatinine, p-urea, p-sodium, p-potassium, p-bicarbonate, p-glucose, p-albumin, p-calcium-ion, p-phosphate, p-uric acid, eGFR, p-cholesterol, p-triglyceride, p-HDL, P-LDL, p-PTH. | 24 hour urine sampling |
| 5b | Week 12 | return of AMBP device |  |
| 6 | Week 16 | b-haemoglobin, b-leukocytes, b-thrombocytes, p-bilirubin, p-ALAT, p-alkaline phosphatase, hsC-reactive protein, p-creatinine, p-urea, p-sodium, p-potassium, p-bicarbonate, p-glucose, p-albumin, p-calcium-ion, p-phosphate, p-uric acid, eGFR. |  |
| 7 | Week 20 | b-haemoglobin, b-leukocytes, b-thrombocytes, p-bilirubin, p-ALAT, p-alkaline phosphatase, hsC-reactive protein, p-creatinine, p-urea, p-sodium, p-potassium, p-bicarbonate, p-glucose, p-albumin, p-calcium-ion, p-phosphate, p-uric acid, eGFR. |  |
| 8a | Week 24 | b-haemoglobin, b-leukocytes, b-thrombocytes, p-bilirubin, p-ALAT, p-alkaline phosphatase, hsC-reactive protein, p-creatinine, p-urea, p-sodium, p-potassium, p-bicarbonate, p-glucose, p-albumin, p-calcium-ion, p-phosphate, p-uric acid, eGFR, p-cholesterol, p-triglyceride, p-HDL, P-LDL, p-parathyroid hormone (PTH) and 1,25-dihydroxy-cholecalciferol (vitamin D3), 25-OH-Vitamin D, p-renin og p-aldosterone. | 24 hour urine sampling |
| 8b | Week 24 | return of AMBP device |  |

Study participants are to collect 24 hour urine samples for analysis of concentrations and total amounts of albumin, creatinine, potassium, sodium and calculation of creatinine clearance at all three visits where pulse wave measurements are made (visits 1, 5a and 8a).

Estimated GFR (eGFR) is routinely performed using the CKD-EPI formula (51).

Re blood samples: At all samplings an amount of blood between 50 and 68 ml of blood will be drawn, depending on purpose of the visit. Analysis is routinely performed in the hospital central laboratory at Herlev Hospital except vitamin D which is performed by Capio Diagnostik and p-renin and aldosterone, which are analyzed at The Dept. of Clinical Physiology, Glostrup Hospital. Both laboratories are usual collaborators for these tests.

Re urine samples: These will be analyzed as routine tests at the central laboratory in Herlev Hospital.

Re frozen tests: Samples will be saved and frozen for later analysis.

Samples are stored in a locked -80°C freezer, which is connected to an alarm system at Herlev Hospital.

### 3.12 Study equipment

Devices which will be used in this study are the ambulatory blood pressure measurement device SpaceLabs 90 217 form SpaceLabs Healthcare, Washington, USA.

For pulse wave recordings we use the applanation tonometer (Millar, Houston, Texas, USA) and the SphygmoCor data handling programme from Atcor Medical, Sydney, Australia.

Office blood pressures are measured using a sphygmanometer.

### 3.13 Practical performance of study

Patients who fulfil the inclusion criteria and who are interested in participating in the study are informed about the study. The written information is handed out and will be thoroughly explained. A form of written informed consent is to be signed by the patient and the informant/investigator. If consent is given, dates for the study days will be given.

Demographic data will be registered.

Randomization: When inclusion criteria are met including the performance of a screening for PW measurements and the run-in period has been completed, patients are randomized to the active treatment arm with eplerenone or the control arm for 24 weeks.

Planning:

A deviation of ± 1 week from the initially planned date for visits is permitted. Collection of 24 hour urine is allowed for at time deviation of ± 3 days with respect to blood sampling. Participants’ visits are planned to be identical in both groups.

Visits are planned as shown below:

| ***Visit number*** | ***Time with reference to inclusion for the individual patient*** | ***Purpose of visit*** | ***Estimated time used for the visit*** |
| --- | --- | --- | --- |
|  | Screening | signing of written informed consent, PW measurement test, ECG, blood sampling, demographic data noted | 1-1½ hours |
| 1a | Week 1 | randomization, PW measurement, AMBP, blood sampling, 24 h urine, study medication handed out | 1 hour |
| 1b | Week 1 | return of AMBP device | 10 minutes |
| 2 | Week 2 | blood sampling, BP, increase in study medication | 30 minutes |
| 3 | Week 4 | blood sampling, BP, study medication handed out | 30 minutes |
| 4 | Week 8 | blood sampling, BP, study medication handed out | 30 minutes |
| 5a | Week 12 | PW measurement, AMBP, blood sampling, 24 h urine, study medication handed out | 1 hour |
| 5b | Week 12 | return of AMBP device | 10 minutes |
| 6 | Week 16 | blood sampling, BP, study medication handed out | 30 minutes |
| 7 | Week 20 | blood sampling, BP, study medication handed out | 30 minutes |
| 8a | Week 24 | PW measurement, AMBP, blood sampling, 24 h urine, study medication and packages returned | 1-1½ hours |
| 8b | Week 24 | return of AMBP device | 10 minutes |

## Procedure for termination of the study

Personal data will be stored for 10 years. It will be destroyed after this. Data will be kept in the Department of Nephrology at Herlev Hospital. Participant’ personal information will be treated with the confidentiality required by the Danish Data Protection Agency (Datatilsynet).

## Statistics

Power calculations have been made based on a minimal clinically relevant difference on the primary end point PWV of 1 m/s. It has been shown that an increase of PWV of this size increases mortality by 39%(19).

From our own data we have a standard deviation of 1.5 on PWV. At a level of significance of 0.05 and power 0.8 a calculation of number of participants for each group should reach 37. We plan to include 40 patients in each group.

Two-sample t test power calculation

**n = 36.3058**

delta = 1

sd = 1.5

sig.level = 0.05

power = 0.8

alternative = two.sided

NOTE: n is number in *each* group

Patients who are with drawn are not replaced.

Differences in PWV will be statistically examined using paired t tests.

The secondary effect parameters will also be examined using this statistic method. For the groups as a whole two sample t test will be applied. If data do not fit the normal distribution they will be transformed.

1. ***Quality control***

Normal procedures for quality control will be followed, see ICH GCP Guidelines. Data will be monitored by The GCP unit at Copenhagen University Hospital, Bispebjerg Hospital, Building 51, 3rd floor, Bispebjerg Bakke 23, DK-2400 Copenhagen NV. As informed consent is given, a permission from the participant for the Danish Medicines Agency and the GCP unit to have access to the patient’s hospital medical files will be sought. A copy of the text is available (in Danish).

## Ethical considerations

*Consequences and advantages of the study*

The study will not directly implicate changes in the treatment of CKD for the individual patient. For the group of patients who suffer from CKD the study aims to reveal possible effects of treatment with eplerenone on arterial stiffness. The result of this could lead to improvement of the prognosis for the CKD patient and the risk of CV disease and death as well as attenuation of progression of uraemia.

*Draw backs and risks of the study*

1: use of time

2: discomfort of having blood samples drawn

3: inconvenience when collecting urine

4: inconvenience when having AMBP device mounted

5: side effects of study medication, including the risk of hyperkalaemia

Re 1: The time used for the patient is quite a lot, but the many visits secure that it is a safe intervention. This is to insure a detection of hyperkalaemia in good time. The many visits also induce a better compliance.

Re 2: At the times of blood sampling a maximum of 68 ml will be drawn. This is done on the first and last visits only. In the remainder of visits, a max. of 54 ml is drawn. This constitutes no risk for the patient.

Re 3: It can be inconvenient to collect urine more often than in a usual outpatient course.

Re 4: Some patients think it tiresome to wear the AMBP device for 24 hours. It is used as a routine examination modality in daily clinical practice. Rarely there are marks from the cuff in the days after the measurements. There are no risks in connection with this examination.

Re 5: Common side effects from the active study drug are seen in more than 1 % and less than 10 % of patients. These can be nausea, diarrhoea, dizziness, hypotension, reduced kidney function and hyperkalaemia. Reduction in kidney function is reversible. The risk of hyperkalaemia is met by the close monitoring plan as described above. The investigational group has good clinical experience in the use of eplerenone in this type of patients.

According to the SPC the following uncommon side effects have been reported in more than 1‰ and less than 1% of patients:

Pyelonephritis, eosinophilia, dehydration, hypercholesterolemia, hypertriglyceridemia, hyponatremia, insomnia, headache, orthostatic hypotension, arterial thrombosis in the leg, atrial fibrillation, myocardial infarction, left ventricular dysfunction, respiratory disorders, disorders in the thorax and mediastinum, pharyngitis, flatulence, vomiting, pruritus, increased perspiration, back ache, leg cramps, asthenia, discomfort, increase in blood urea nitrogen, increased plasma creatinine.

## Adverse events and side effects

Registration and reporting of side effects follow the given laws. In the following the document of referral is in Danish: *Bekendtgørelsen for kliniske forsøg med lægemidler på mennesker* (The notice for clinical studies using pharmaceutical drugs on humans). Suspected and unexpected adverse reactions (SUSAR), that are either lethal or life threatening will be reported to the Danish Medicines Agency within 7 days of notice to the sponsor-investigator and at the latest at day 8 after this the Agency will be informed of all actions taken on the follow up. All unexpected and serious suspected side effects will be reported to the Danish Medicines Agency at the latest 15 days after it has come to the attention of the sponsor-investigator. Serious adverse reactions (SAR) will be reported on a yearly basis to the Regional Ethics Committee. Events will be recorded on the patients’ CRF excepting laboratory results that lie outside the reference values but where no action is taken to either change the study medication or institute treatment. Common viral diseases such as colds or flus will not be reported. Finally uncomplicated urinary tract infections will not be reported.

Events and side effects will be reported in the final report to the Danish Medicines Agency after termination of the study. Events and side effects will be registered from the time of initial dosing of study medication until withdrawal.

In case of unacceptable side effects the participant will be withdrawn from the study. The patient will continue in the out patients clinic of nephrology as long as this is required.

The study can be terminated before time according to the sponsor-investigator’s judgement.

## Resume

Patients with CKD have a poor prognosis primarily due to cardiovascular disease. The cardiovascular risk can be assessed by measurements of arterial stiffness. A decrease in stiffness has been shown to decrease the risk of cardiovascular disease as well as death. Most of the CKD population also have hypertension and control of blood pressure is one of the cornerstones in inhibition of disease progression. Using drugs that specifically block the renin-angiotensin-system for blood pressure control has been shown to have beneficial impact on inhibition of progression beyond that of achieved blood pressure control. It has been reported that inhibition of the hormone aldosterone has a positive effect on survival in patients with heart failure, hypertension and diabetic as well as on diabetic nephropathy.

This study undertakes the investigation of the influence on arterial stiffness of adding an aldosterone receptor inhibitor to the medication CKD patients are already taking. Besides the primary end point which is PWV, arterial stiffness is also quantified thorough ambulatory blood pressure measurements.

The study is a prospective, randomized, controlled, parallel group, phase IV study. It is investigator initiated. Participants are to be found amongst patients already attending the outpatient clinics at the departments of nephrology at Herlev Hospital or Rigshospitalet, Copenhagen, Denmark. All visits will take place at Herlev Hospital. The study will be performed only after permission has been granted by the regional ethics committee, Danish Medicines Agency and Danish Data Protection Agency. Monitoring will be done by The GCP unit at Copenhagen University Hospital. The study has been supported by grants from private funds and Department of Nephrology, Herlev Hospital.

## Information of the patient and informed consent

Potential study participants are patients with CKD stages 3-4, who are undergoing treatment in the outpatient clinics at the departments of nephrology at either Herlev Hospital or Rigshospitalet at the time of enrolment.

*Guidelines for oral information and obtaining written informed consent*

**Before consultation**

Eligible patients are informed of the study at a usual control visit in the clinic. If the patient is interested in more information a time and place is set for the full information to be given. Beforehand the patient will be informed that the visit will be in the interest of informing them about a clinical study. He or she will be made aware of their right to bring an assessor. It is explained that the patient is allowed to drop out of the study whenever he or she wants to with out having to explain why and that such a drop out will not influence on their further possibilities of treatment in the clinics. A time period of 1 week is allowed for consideration after information has been given. Along with written information on the study patients are also given the central ethics committee’s paper on rights for patients who participate in clinical studies (”**Forsøgspersonens rettigheder i et biomedicinsk forskningsprojekt”).**

**Consultation**

Information will take place in quiet undisturbed surroundings at a given time. Information is given by a doctor, who is investigator in the study. The patient and his assessor are informed orally and then given the written information to read. Questions are to be answered. The patient is explained that it is possible to have an extra consultation regarding information before accepting to participate. If the patient accepts he is asked to sign the forms of consent within a week after. The patient is informed that he is most welcome to contact the doctors in the study group.

**Before enrolment**

The patient has to sign the forms of consent before the screening procedure is carried out. Screening consists of PW measurement and ECG to confirm sinus rhythm. If laboratory results show 25-OH-Vitamin D < 75 nmol/L this deficiency is treated following the present guidelines (52) for at least 4 weeks prior to randomization and PW measurements. Blood pressure is also evaluated for 4 weeks before start of study treatment. The participant receives a photocopy of the signed inform consent and a calendar with the dates of all visits plotted.

## Publication and reporting to authorities

The results, be they negative or positive, will be made publicly available. The study will be submitted to an international peer-reviewed journal. The article will be written by Lene Boesby. Co-authors will be Thomas Elung-Jensen, Svend Strandgaard and Anne-Lise Kamper.

After termination of the study it is the duty of the sponsor-investigator to inform the Danish Medicines Agency and the Regional Ethics Committee that the study has been completed.

## Insurance

Participants are covered by “Patientforsikringen” during the study.

## Economy

The study is initiated by sponsor and investigator. Funding is provided from private and public funds as well as the Department of Nephrology, Herlev Hospital. The study drug is handed out by the sponsor-investigator free of charge for participants. Expenses for this are held by the Department of Nephrology, Herlev Hospital. The sponsor-investigator has ownership of all data generated form the study. Salaries for the principal investigator are paid by funding and amount follows the collective agreement at the time of payment.

The SphygmoCor device and tonometer is sponsored by Toyota-FONDEN, Civilingeniør Frode V. Nyegaard and Hustrus Fond and Grosserer LF Foghts Fond. Neither of these have economic interests in the study. The ambulatory blood pressure measuring devices are sponsored by the Department of Nephrology, Herlev Hospital and Helen and Ejnar Bjørnows Fond.

**Reference List**

**(1) Hallan SI, Coresh J, Astor BC, Asberg A, Powe NR, Romundstad S, et al. International comparison of the relationship of chronic kidney disease prevalence and ESRD risk. J Am Soc Nephrol 2006 Aug;17(8):2275-84.**

**(2) The Danish Society of Nephrology. Danish National Registry Report Annual Report 2007. 2008.**

**(3) Randomised placebo-controlled trial of effect of ramipril on decline in glomerular filtration rate and risk of terminal renal failure in proteinuric, non-diabetic nephropathy. The GISEN Group (Gruppo Italiano di Studi Epidemiologici in Nefrologia). Lancet 1997 Jun 28;349(9069):1857-63.**

**(4) Kamper AL, Strandgaard S, Leyssac PP. Effect of enalapril on the progression of chronic renal failure. A randomized controlled trial. Am J Hypertens 1992 Jul;5(7):423-30.**

**(5) Werner C, Baumhakel M, Teo KK, Schmieder R, Mann J, Unger T, et al. RAS blockade with ARB and ACE inhibitors: current perspective on rationale and patient selection. Clin Res Cardiol 2008 Jul;97(7):418-31.**

**(6) Dahlof B, Devereux RB, Kjeldsen SE, Julius S, Beevers G, de FU, et al. Cardiovascular morbidity and mortality in the Losartan Intervention For Endpoint reduction in hypertension study (LIFE): a randomised trial against atenolol. Lancet 2002 Mar 23;359(9311):995-1003.**

**(7) Barnett AH, Bain SC, Bouter P, Karlberg B, Madsbad S, Jervell J, et al. Angiotensin-receptor blockade versus converting-enzyme inhibition in type 2 diabetes and nephropathy. N Engl J Med 2004 Nov 4;351(19):1952-61.**

**(8) Balamuthusamy S, Srinivasan L, Verma M, Adigopula S, Jalandara N, Hathiwala S, et al. Renin angiotensin system blockade and cardiovascular outcomes in patients with chronic kidney disease and proteinuria: a meta-analysis. Am Heart J 2008 May;155(5):791-805.**

**(9) Pitt B, Zannad F, Remme WJ, Cody R, Castaigne A, Perez A, et al. The effect of spironolactone on morbidity and mortality in patients with severe heart failure. Randomized Aldactone Evaluation Study Investigators. N Engl J Med 1999 Sep 2;341(10):709-17.**

**(10) Pitt B, White H, Nicolau J, Martinez F, Gheorghiade M, Aschermann M, et al. Eplerenone reduces mortality 30 days after randomization following acute myocardial infarction in patients with left ventricular systolic dysfunction and heart failure. J Am Coll Cardiol 2005 Aug 2;46(3):425-31.**

**(11) Chrysostomou A, Pedagogos E, MacGregor L, Becker GJ. Double-blind, placebo-controlled study on the effect of the aldosterone receptor antagonist spironolactone in patients who have persistent proteinuria and are on long-term angiotensin-converting enzyme inhibitor therapy, with or without an angiotensin II receptor blocker. Clin J Am Soc Nephrol 2006 Mar;1(2):256-62.**

**(12) Tylicki L, Rutkowski P, Renke M, Larczynski W, Aleksandrowicz E, Lysiak-Szydlowska W, et al. Triple pharmacological blockade of the renin-angiotensin-aldosterone system in nondiabetic CKD: an open-label crossover randomized controlled trial. Am J Kidney Dis 2008 Sep;52(3):486-93.**

**(13) Epstein M. Aldosterone as a mediator of progressive renal disease: pathogenetic and clinical implications. Am J Kidney Dis 2001 Apr;37(4):677-88.**

**(14) Lacolley P, Labat C, Pujol A, Delcayre C, Benetos A, Safar M. Increased carotid wall elastic modulus and fibronectin in aldosterone-salt-treated rats: effects of eplerenone. Circulation 2002 Nov 26;106(22):2848-53.**

**(15) Kiyomoto H, Rafiq K, Mostofa M, Nishiyama A. Possible underlying mechanisms responsible for aldosterone and mineralocorticoid receptor-dependent renal injury. J Pharmacol Sci 2008 Dec;108(4):399-405.**

**(16) Toussaint ND, Kerr PG. Vascular calcification and arterial stiffness in chronic kidney disease: implications and management. Nephrology (Carlton ) 2007 Oct;12(5):500-9.**

**(17) Blacher J, Guerin AP, Pannier B, Marchais SJ, Safar ME, London GM. Impact of aortic stiffness on survival in end-stage renal disease. Circulation 1999 May 11;99(18):2434-9.**

**(18) Guerin AP, Blacher J, Pannier B, Marchais SJ, Safar ME, London GM. Impact of aortic stiffness attenuation on survival of patients in end-stage renal failure. Circulation 2001 Feb 20;103(7):987-92.**

**(19) Blacher J, Asmar R, Djane S, London GM, Safar ME. Aortic pulse wave velocity as a marker of cardiovascular risk in hypertensive patients. Hypertension 1999 May;33(5):1111-7.**

**(20) McEniery CM, Yasmin, Hall IR, Qasem A, Wilkinson IB, Cockcroft JR. Normal vascular aging: differential effects on wave reflection and aortic pulse wave velocity: the Anglo-Cardiff Collaborative Trial (ACCT). J Am Coll Cardiol 2005 Nov 1;46(9):1753-60.**

**(21) Klingbeil AU, John S, Schneider MP, Jacobi J, Weidinger G, Schmieder RE. AT1-receptor blockade improves augmentation index: a double-blind, randomized, controlled study. J Hypertens 2002 Dec;20(12):2423-8.**

**(22) London GM, Asmar RG, O'Rourke MF, Safar ME. Mechanism(s) of selective systolic blood pressure reduction after a low-dose combination of perindopril/indapamide in hypertensive subjects: comparison with atenolol. J Am Coll Cardiol 2004 Jan 7;43(1):92-9.**

**(23) London GM, Pannier B, Vicaut E, Guerin AP, Marchais SJ, Safar ME, et al. Antihypertensive effects and arterial haemodynamic alterations during angiotensin converting enzyme inhibition. J Hypertens 1996 Sep;14(9):1139-46.**

**(24) Suzuki H, Nakamoto H, Okada H, Sugahara S, Kanno Y. A selective angiotensin receptor antagonist, Valsartan, produced regression of left ventricular hypertrophy associated with a reduction of arterial stiffness. Adv Perit Dial 2003;19:59-66.**

**(25) Dolan E, Thijs L, Li Y, Atkins N, McCormack P, McClory S, et al. Ambulatory arterial stiffness index as a predictor of cardiovascular mortality in the Dublin Outcome Study. Hypertension 2006 Mar;47(3):365-70.**

**(26) Dolan E, Li Y, Thijs L, McCormack P, Staessen JA, O'Brien E, et al. Ambulatory arterial stiffness index: rationale and methodology. Blood Press Monit 2006 Apr;11(2):103-5.**

**(27) Hansen TW, Staessen JA, Torp-Pedersen C, Rasmussen S, Li Y, Dolan E, et al. Ambulatory arterial stiffness index predicts stroke in a general population. J Hypertens 2006 Nov;24(11):2247-53.**

**(28) Kikuya M, Staessen JA, Ohkubo T, Thijs L, Metoki H, Asayama K, et al. Ambulatory arterial stiffness index and 24-hour ambulatory pulse pressure as predictors of mortality in Ohasama, Japan. Stroke 2007 Apr;38(4):1161-6.**

**(29) Li Y, Dolan E, Wang JG, Thijs L, Zhu DL, Staessen JA, et al. Ambulatory arterial stiffness index: determinants and outcome. Blood Press Monit 2006 Apr;11(2):107-10.**

**(30) Li Y, Wang JG, Dolan E, Gao PJ, Guo HF, Nawrot T, et al. Ambulatory arterial stiffness index derived from 24-hour ambulatory blood pressure monitoring. Hypertension 2006 Mar;47(3):359-64.**

**(31) Willum-Hansen T, Staessen JA, Torp-Pedersen C, Rasmussen S, Thijs L, Ibsen H, et al. Prognostic value of aortic pulse wave velocity as index of arterial stiffness in the general population. Circulation 2006 Feb 7;113(5):664-70.**

**(32) Agata J, Nagahara D, Kinoshita S, Takagawa Y, Moniwa N, Yoshida D, et al. Angiotensin II receptor blocker prevents increased arterial stiffness in patients with essential hypertension. Circ J 2004 Dec;68(12):1194-8.**

**(33) Anan F, Takahashi N, Ooie T, Yufu K, Hara M, Nakagawa M, et al. Effects of valsartan and perindopril combination therapy on left ventricular hypertrophy and aortic arterial stiffness in patients with essential hypertension. Eur J Clin Pharmacol 2005 Jul;61(5-6):353-9.**

**(34) Asmar R. Effect of antihypertensive agents on arterial stiffness as evaluated by pulse wave velocity: clinical implications. Am J Cardiovasc Drugs 2001;1(5):387-97.**

**(35) Ichihara A, Kaneshiro Y, Sakoda M, Takemitsu T, Itoh H. Add-on amlodipine improves arterial function and structure in hypertensive patients treated with an angiotensin receptor blocker. J Cardiovasc Pharmacol 2007 Mar;49(3):161-6.**

**(36) Karalliedde J, Smith A, DeAngelis L, Mirenda V, Kandra A, Botha J, et al. Valsartan improves arterial stiffness in type 2 diabetes independently of blood pressure lowering. Hypertension 2008 Jun;51(6):1617-23.**

**(37) Lacourciere Y, Beliveau R, Conter HS, Burgess ED, Lepage S, Pesant Y, et al. Effects of perindopril on elastic and structural properties of large arteries in essential hypertension. Can J Cardiol 2004 Jun;20(8):795-9.**

**(38) Topouchian J, El FR, Pannier B, Wang S, Zhao F, Smetana K, et al. Arterial stiffness and pharmacological interventions--the TRanscend arterial stiffNess Substudy (TRANS study). Vasc Health Risk Manag 2007;3(4):381-7.**

**(39) Schjoedt KJ, Rossing K, Juhl TR, Boomsma F, Tarnow L, Rossing P, et al. Beneficial impact of spironolactone on nephrotic range albuminuria in diabetic nephropathy. Kidney Int 2006 Aug;70(3):536-42.**

**(40) Savoia C, Touyz RM, Amiri F, Schiffrin EL. Selective mineralocorticoid receptor blocker eplerenone reduces resistance artery stiffness in hypertensive patients. Hypertension 2008 Feb;51(2):432-9.**

**(41) Gallagher D, Adji A, O'Rourke MF. Validation of the transfer function technique for generating central from peripheral upper limb pressure waveform. Am J Hypertens 2004 Nov;17(11 Pt 1):1059-67.**

**(42) Frimodt-Moller M, Nielsen AH, Kamper AL, Strandgaard S. Pulse-wave morphology and pulse-wave velocity in healthy human volunteers: examination conditions. Scand J Clin Lab Invest 2006;66(5):385-94.**

**(43) Frimodt-Moller M, Nielsen AH, Kamper AL, Strandgaard S. Reproducibility of pulse-wave analysis and pulse-wave velocity determination in chronic kidney disease. Nephrol Dial Transplant 2008 Feb;23(2):594-600.**

**(44) Savage MT, Ferro CJ, Pinder SJ, Tomson CR. Reproducibility of derived central arterial waveforms in patients with chronic renal failure. Clin Sci (Lond) 2002 Jul;103(1):59-65.**

**(45) Wilkinson IB, Fuchs SA, Jansen IM, Spratt JC, Murray GD, Cockcroft JR, et al. Reproducibility of pulse wave velocity and augmentation index measured by pulse wave analysis. J Hypertens 1998 Dec;16(12 Pt 2):2079-84.**

**(46) Stergiou GS, Efstathiou SP, Argyraki CK, Gantzarou AP, Roussias LG, Mountokalakis TD. Clinic, home and ambulatory pulse pressure: comparison and reproducibility. J Hypertens 2002 Oct;20(10):1987-93.**

**(47) Hansen KW, Poulsen PL, Christiansen JS, Mogensen CE. Determinants of 24-h blood pressure in IDDM patients. Diabetes Care 1995 Apr;18(4):529-35.**

**(48) Dechering DG, van der Steen MS, Adiyaman A, Thijs L, Deinum J, Li Y, et al. Reproducibility of the ambulatory arterial stiffness index in hypertensive patients. J Hypertens 2008 Oct;26(10):1993-2000.**

**(49) Bang LE, Christensen KL, Hansen KW, Skov K, Wiinberg N. Diagnostisk blodtryksmåling - på døgnbasis, hjemme og i konsultationen. 2006.**

**(50) K-DOQI guidelines. 2007. 24-4-2007.
Ref Type: Internet Communication**

**(51) Levey AS, Stevens LA, Schmid CH, Zhang YL, Castro AF, III, Feldman HI, et al. A new equation to estimate glomerular filtration rate. Ann Intern Med 2009 May 5;150(9):604-12.**

**(52) Selskab DN. Henrik Daugaard, James Heaf, Preben Joffe, Ewa Lewin, S°ren Madsen, Marianne Rix, Klaus Olgaard: Forstyrrelser i knogle og mineralomsμtningen ved kronisk nyresygdomûguidelines for diagnostik og behandling. K/DOQI guidelines konverteret til danske forhold Dansk Nefrologisk Selskab 2005.**
